# Initiation and continuation of pharmacological therapies in patients hospitalized for heart failure in Japan

**DOI:** 10.1038/s41598-024-60011-y

**Published:** 2024-04-20

**Authors:** Suguru Okami, Coralie Lecomte, Hanaya Raad, Mireia Aguila, Zuzana Mohrova, Makiko Takeichi, Takanori Tsuchiya, Christoph Ohlmeier, Thomas Evers, Alexander Michel

**Affiliations:** 1 Medical Affairs & Pharmacovigilance, Bayer Yakuhin Ltd., Breeze Tower, 2-4-9 Umeda, Kita-Ku, Osaka, 530-0001 Japan; 2 Aetion Inc., 5 Penn Plaza, New York, USA; 3 Market Access & Public Affairs, Bayer Yakuhin, Ltd, 2-4-9 Umeda, Kita-Ku, Osaka, Japan; 4 Integrated Evidence Generation & Business Innovation, Bayer AG, 13342 Berlin, Germany; 5 Integrated Evidence Generation & Business Innovation, Bayer AG, 42096 Wuppertal, Germany; 6 Integrated Evidence Generation & Business Innovation, Bayer Consumer Care AG, Peter Merian Straße 84, 4052 Basel, Switzerland

**Keywords:** Cardiology, Drug therapy, Epidemiology

## Abstract

Currently, the utilization patterns of medications for heart failure (HF) after worsening HF events remain unelucidated in Japan. Here, we conducted a retrospective cohort study evaluating the changes in HF drug utilization patterns in 6 months before and after hospitalizations for HF. The adherence to newly initiated HF medications was evaluated based on the proportion of days covered (PDC) and persistence as continuous treatment episodes among new users. The study included 9091 patients hospitalized for HF between January 2016 and September 2019, including 2735 (30.1%) patients who were newly prescribed at least one HF medication after hospitalization. Despite increases in the use of foundational HF therapy (beta-blockers, angiotensin-converting-enzyme inhibitors/angiotensin receptor blockers, or mineralocorticoid receptor antagonists), 35.6% and 7.6% of patients were treated with the HF foundational monotherapy or diuretics alone after hospitalization, respectively. The mean PDC of newly initiated HF medications ranged from 0.57 for thiazide diuretics to 0.77 for sodium-glucose cotransporter-2 inhibitors. Continuous use of HF medications during the first year after initiation was observed in 30–60% of patients. The mean PDC and one-year continuous HF medication use were consistently lower in patients aged ≥ 75 years and in patients with a history of HF hospitalization for all HF medication classes except for tolvaptan and digoxin. Despite the guideline recommendations of HF pharmacotherapy, both treatment and adherence were suboptimal after HF hospitalization, especially in vulnerable populations such as older patients and those with prior HF hospitalizations.

## Introduction

Along with an increasing aging population, the prevalence of heart failure (HF) is rising globally, leading to a significant medical and socioeconomic burden^1^. It has been recognized as a global pandemic, with an estimated 64.3 million people suffering from HF worldwide in 2017^2^. In Japan, the number of patients with left ventricular dysfunction was estimated to 979,000 in 2005 (0.8% of the total population), which was projected to increase gradually, reaching 1.3 million by 2030^3^.

Hospitalization for HF indicates a deteriorating prognosis for re-hospitalization and mortality^4,5^. Despite improved outcomes owing to significant advances in therapies, the risks of hospitalization for HF and mortality remain high^6–8^. A study from the Japanese registry of acute decompensated HF (JROADHF) reported incidence rates (per 100 person-years) for cardiovascular death, HF hospitalization, and all-cause death of 7.1, 21.1, and 14.9, respectively^9^. The risks of all-cause death and HF readmission were even higher in patients with a history of repeated hospitalization for HF^10^. Guideline-directed medical therapy (GDMT) has proven its efficacy in reducing the risks of hospitalization for HF and cardiovascular mortality in patients with HF^11–13^. In the real-world clinical settings^14,15^ however, studies have reported the underuse of GDMT after worsening HF events such as hospitalization for the decompensation of HF or the outpatient treatment with intravenous diuretics for fluid overload^16–19^.

Despite its importance due to the high risk of HF readmission, comorbid burden, and the risk of HF decompensation events, there is currently limited information on changes in HF medications and the persistence of the treatment initiated after worsening HF events in Japan. Retrospective analyses of large-scale registries of patients hospitalized for HF reported the changes in HF medication patterns from admission to discharge; however, the longitudinal data on treatment continuations were lacking^9,20^. In a multinational study, patterns of titration and discontinuation of newly initiated GDMT after worsening HF events were reported; however, the study lacked data on other HF medications, such as diuretics and digoxin^21^. Furthermore, considering the increasing proportions of older HF patients^22^ and worsened prognosis of patients with repeated hospitalizations for HF^10,23,24^, it is necessary to elucidate HF drug utilization patterns specifically in these populations with high medical and socioeconomic burden associated with HF^22,25^.

Herein, we aimed to provide comprehensive insights on HF drug utilization patterns, including adherence to newly initiated medications and clinical outcomes following an HF decompensation event in real-world patients, using an extensive nationwide hospital database in Japan reflecting a range of clinical settings.

## Methods

### Study design, data source, and patient selection

We conducted a retrospective cohort study using a nationwide hospital database in Japan. The study utilized the data extracted from the Real-World Database (RWD) maintained by the Health, Clinic and Education Information Evaluation Institute with technical support from Real World Data Co. Ltd. (Kyoto, Japan). The RWD contains electronic medical records and diagnosis procedure combination (DPC) data^26^ linked to medical claims collected from > 200 medical institutions in Japan, covering most of the geographic regions and all age groups in the country. This database contains procedure records (admission, discharge, outpatient, and DPC), laboratory results, prescriptions, and hospital-based diagnoses following the International Classification of Diseases 10th Revision (ICD-10) codes. As of October 2021, the RWD collected medical records from > 20 million patients. The study period was from January 1, 2016 until October 31, 2021.

**Figure 1:**
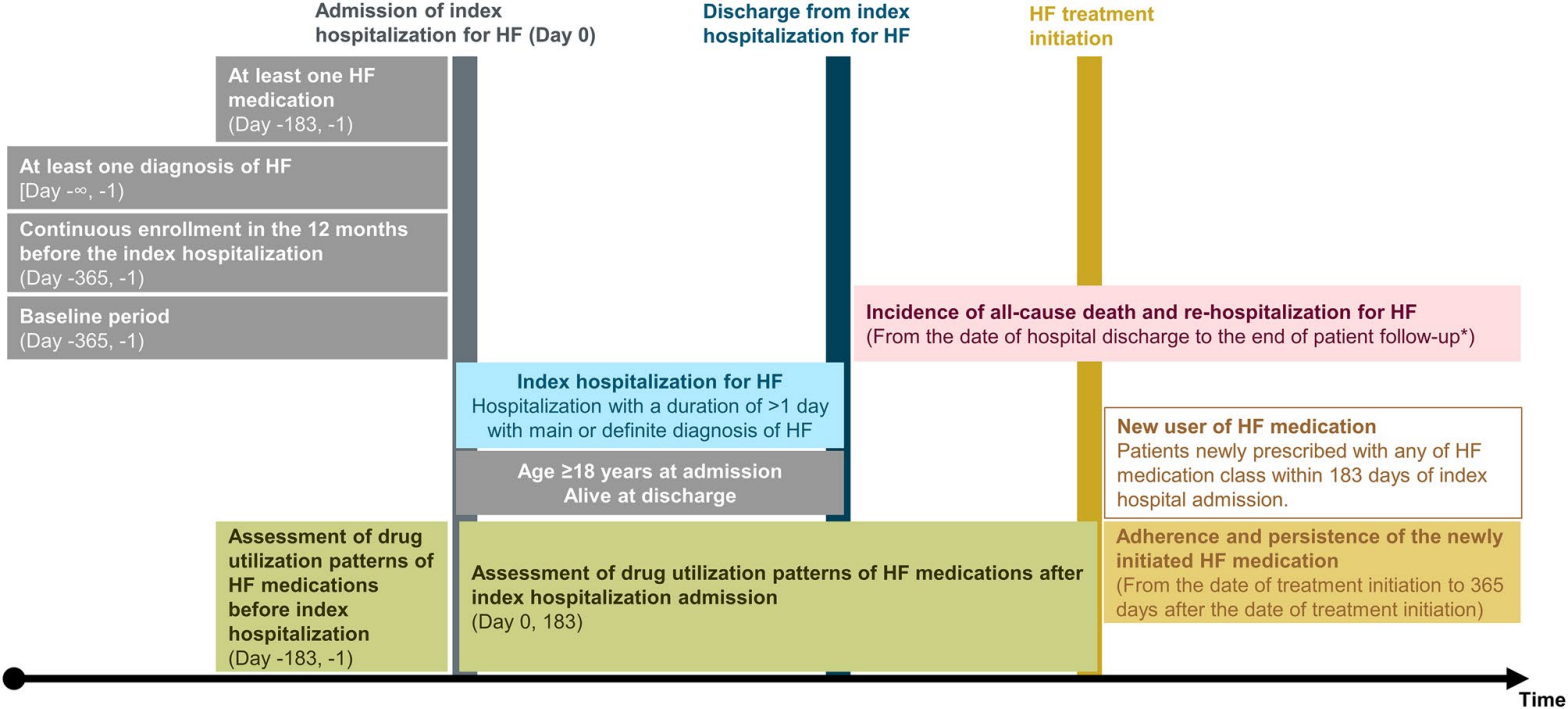
Summary of study design. *Patients were followed up until the timing of death, emigration from dataset, or the end of study period (October 31, 2021), whichever came first. *HF* heart failure.

Figure 1 shows the summary of study design. Patients were identified based on a record of hospitalization for HF, defined based on one primary or definite diagnosis of HF (ICD-10 I50.x or I11.0) during the hospitalization with a duration of > 1 day, occurring between January 1, 2016, and September 30, 2019 (the identification period). We employed the record of hospitalization for HF to identify patients with worsening HF, and not based on the decongested symptoms and the use of intravenous diuretics, since the  record of hospitalization with a primary or discharge diagnosis of HF could be more regorous criteria than that based on symptoms. We selected patients aged ≥ 18 years and with at least one diagnosis of HF and one prescription of HF medication before the index hospitalization. The study did not include patients dying during the index hospitalization and those without continuous enrollment in the database for 12 months before the index hospitalization. Patients were followed up until death, end of activity in the dataset, or at the end of the study period (October 31, 2021), whichever came first.

### Study variables and outcomes

The baseline characteristics, evaluated over a 12-month period before the index hospitalization, included demographics, comorbidities, procedures, comedications, laboratory data, and prior hospitalization for HF. The laboratory data and procedure information were also summarized during the index hospitalization for HF. If more than one laboratory data was available, the first recorded data after hospital admission was used. The records of clinical events including all-cause mortality and hospitalization for HF were collected during the follow-up period after discharge. The variables used to define eligibility, comorbidities and clinical events collected in the study can be found in Supplementary Table S1. The HF medications analyzed included beta-blockers, angiotensin-converting-enzyme inhibitors (ACEis), angiotensin-receptor blockers (ARBs), mineralocorticoid receptor antagonists (MRAs), sodium-glucose cotransporter-2 inhibitors (SGLT-2is), digoxin/ digitoxin, loop and thiazide diuretics, and tolvaptan. These medications were identified based on the anatomical therapeutic chemical classification system codes (Supplementary Table S2). As ivabradine (approved in September, 2019) and angiotensin receptor blocker neprilysin inhibitor (ARNI; approved in June, 2020) were unavailable in Japan during the identification period, these were not included.

### Statistical analyses

Continuous variables were reported as mean, standard deviation (SD), median, and interquartile range (IQR). Frequency and percentages were used to document categorical measures. Missing data were not imputed. The incidence rates per 1,000 person-years for clinical events were reported with 95% confidence intervals (CIs). The Kaplan–Meier curves of clinical events were computed using all available follow-up data during the study period. The drug utilization patterns of HF medications were summarized for the 6-month period before the index hospitalization and the period from the index date of hospital admission to 6 months post-discharge, separately. To be considered as any HF medication, a minimum of 30 days duration was required for a valid treatment episode. The new users of HF medications were identified from the time of the index hospitalization until 6 months post-discharge after confirming that these patients were not prescribed the HF medications of interest during the 6 months before the index hospitalization. The proportion of days covered (PDC) was summarized as a measure of adherence from the start of new HF medications until one year after the initiation. It was calculated by dividing the number of days covered by treatment episodes by the number of days the patient was retained in the analysis cohort^27^. The numbers and percentages of new users with a continuous treatment episode of one year after starting HF medications were reported as measures of persistence to the treatment. Treatment discontinuation was defined as not being prescribed the same treatment after 30 days following the end of the last treatment episode. For these analyses, patients were required to have observability for 365 days after starting HF medications. A sensitivity analysis was performed by applying a 60-day gap to determine treatment discontinuation. The analyses were conducted in all patients and subgroups stratified based on age (< 75 or ≥ 75 years), and based on history of hospitalization for HF within one year before the index hospitalization. Statistical analyses were performed using the Aetion Evidence Platform (Aetion® Substantiate). This study followed the STROBE (Strengthening the Reporting of Observational Studies in Epidemiology) statement^28^, which is elaborated in Supplementary Table S3.

### Ethics statement

The ethics committee approval was not required because this study only used already anonymized and deidentified secondary data. In Japan, ethical approval and informed consent do not apply to the use of de-identified secondary data in accordance with the Ethical Guidelines for Medical and Health Research Involving Human Subjects^29^. The use of deidentified data was in accordance with local regulations including the Personal Information Protection Law. This study was conducted in compliant to the Declaration of Helsinki.

## Results

### Patient characteristics

**Figure 2:**
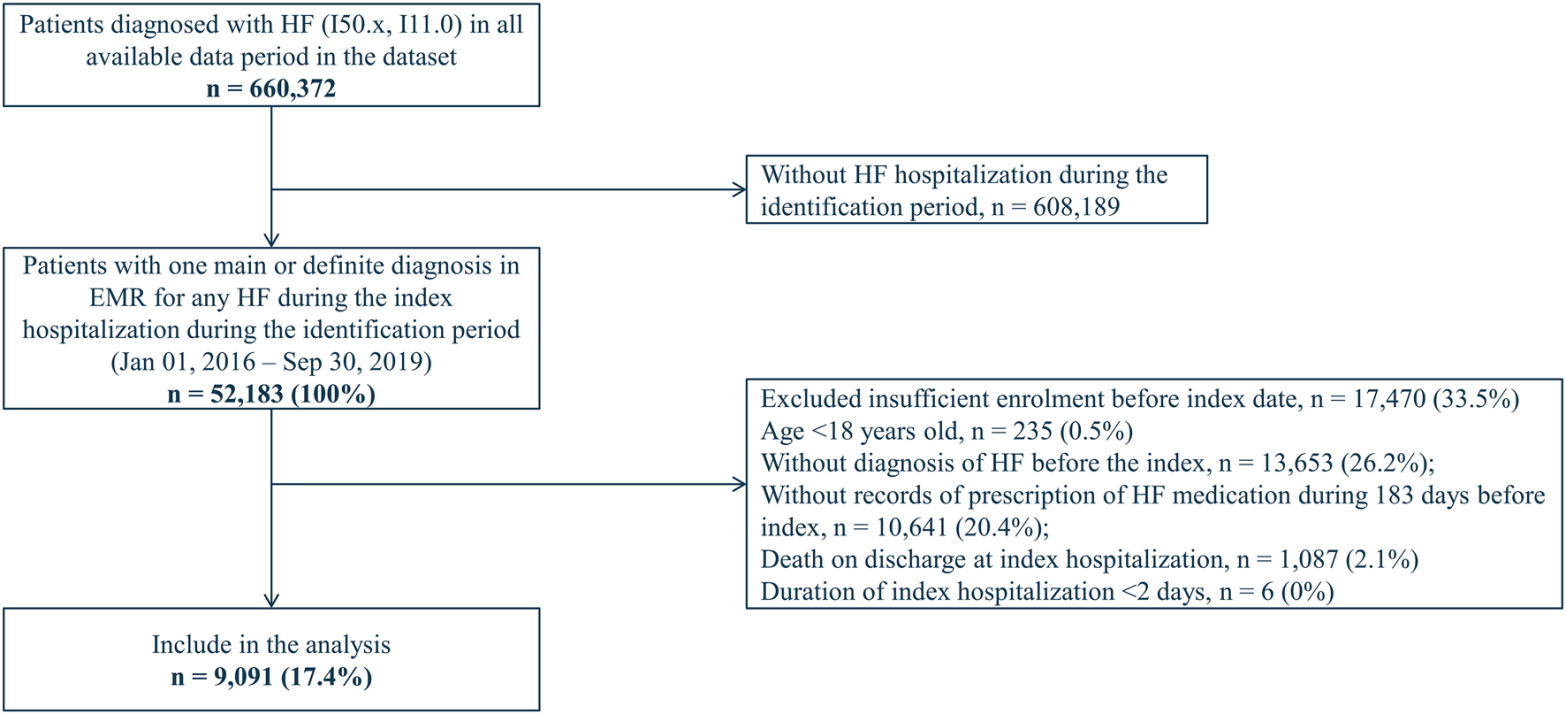
Flow diagram of patient inclusion in the study. *HF* heart failure, *EMR* electronic medical records.

Out of a total of 660,372 patients diagnosed with HF recorded in the dataset, 52,183 patients were identified with a record of hospitalization for HF during the identification period. After applying eligibility criteria, 9091 (17.4%) patients were included in the analysis (Fig. 2). The median length of follow-up was 722 days. The median (IQR) duration of the index hospitalization was 18 (10–34) days and 25% of patients received cardiac rehabilitation (Supplementary Table S4). One-year incidence rates (95% CI) of deaths and re-hospitalizations for HF post-discharge were 151.1 (142.2–160.0) and 195.8 (185.2–206.3) per 1000 person-years, respectively (Supplementary Fig. S1). After the index hospital admission, initiation of an additional HF drug class was observed in 2735 (30.1%) patients. Of these, the vast majority (n = 2,147 [78.5%]) of patients initiated the medication during the index hospitalization. While the proportions of new users were similar between patients with age < 75 years and ≥ 75 years (30.6 vs. 29.9%), it was lower in patients with a history of HF hospitalization compared to those without prior HF hospitalization (21.1 vs. 31.7%).

Table 1 shows the patient characteristics in the overall study population, new users, and non-new users of HF medications. In the overall study population, the mean age was 77.6 years (70.0% of patients were ≥ 75 years old) and 55.1% were male. In the year before the index hospitalization, 40.3% did not have a record of visiting a cardiologist and 15.1% of patients had a history of HF hospitalization. Further, 86.2, 55.9, 56.9, and 39.3% of patients had hypertension, chronic kidney disease (CKD), ischemic heart disease, and atrial fibrillation, respectively. At baseline, the mean ± SD brain natriuretic peptide (BNP) level was 336.6 ± 509.9 pg/mL. Table 2 summarizes the characteristics of new users by each medication class. The patient characteristics of new users were similar to those of non-new users, except for BNP levels (374.9 vs. 319.7 pg/mL; being higher in new users vs. non-new users) and patients with a history of hospitalization for HF (10.6 vs. 17.0%; being lower in new users vs. non-new users). The new users with a history of hospitalization for HF were more comorbid and with higher baseline BNP levels compared to other subgroups. (Supplementary Table S5).

**Table 1:** Baseline characteristics in all patients, including new and non-new users of heart failure treatment

	Overall (N = 9091)	New users (N = 2735)	Non-new users (N = 6356)
Age (years)			
Mean±SD	77.6 ± 10.0	77.4 ± 10.3	77.6±9.9
Age group, n (%)			
< 40	56 (0.6)	19 (0.7)	37 (0.6)
40–64	841 (9.3)	253 (9.3)	588 (9.3)
65–74	1827 (20.1)	561 (20.5)	1266 (19.9)
≥75	6367 (70.0)	1902 (69.5)	4465 (70.2)
Gender, male, n (%)	5007 (55.1)	1477 (54.0)	3530 (55.5)
BNP (pg/mL)			
Patients with recorded BNP values, n (%)	6534 (71.9)	2008 (73.4)	4526 (71.2)
Mean±SD	336.6 ± 509.9	374.9 ± 499.3	319.7 ± 513.7
BNP/NT-proBNP category, n (%)			
BNP ≤ 100 or NT-proBNP ≤ 400 pg/mL	2302 (25.3)	541 (19.8)	1761 (27.7)
100 < BNP ≤ 200 or 400 < NT-proBNP ≤ 900 pg/mL	1366 (15.0)	413 (15.1)	953 (15.0)
200 < BNP ≤ 300 or 900 < NT-proBNP ≤ 2000 pg/mL	991 (10.9)	340 (12.4)	651 (10.2)
BNP > 300 or NT-proBNP ≥ 2000 pg/mL	2548 (28.0)	913 (33.4)	1635 (25.7)
Missing	1884 (20.7)	528 (19.3)	1356 (21.3)
Comorbidity, n (%)			
Hypertension	7837 (86.2)	2365 (86.5)	5472 (86.1)
Chronic kidney disease	5082 (55.9)	1585 (58.0)	3568 (56.1)
Ischemic heart disease	5174 (56.9)	1539 (56.3)	3635 (57.2)
Atrial fibrillation	3571 (39.3)	1154 (42.2)	2417 (38.0)
Diabetes mellitus	3145 (34.6)	954 (34.9)	2191 (34.5)
Stroke	1906 (21.0)	569 (20.8)	1337 (21.0)
Myocardial infarction	1798 (19.8)	496 (18.1)	1302 (20.5)
Chronic obstructive pulmonary disease	1711 (18.8)	516 (18.9)	1195 (18.8)
Anemia	3120 (34.3)	909 (33.2)	2211 (34.8)
Hyperkalemia	1029 (11.3)	304 (11.1)	725 (11.4)
Hypotension	330 (3.6)	54 (2.0)	276 (4.3)
Cardiovascular procedure, n (%)			
Cardiac resynchronization therapy	215 (2.4)	65 (2.4)	150 (2.4)
Implantable cardioverter defibrillator	41 (0.5)	16 (0.6)	25 (0.4)
Having a history of prior hospitalization for HF*, n (%)	1372 (15.1)	290 (10.6)	1082 (17.0)
HF treatments before the index date**, n (%)			
ACEi	1291 (14.2)	376 (13.7)	915 (14.4)
ARB	3519 (38.7)	1075 (39.3)	2444 (38.5)
MRA	1765 (19.4)	461 (16.9)	1304 (20.5)
Beta-blockers	3801 (41.8)	1058 (38.7)	2743 (43.2)
SGLT-2i	198 (2.2)	59 (2.2)	139 (2.2)
Digoxin/digitoxin	424 (4.7)	141 (5.2)	283 (4.5)
Loop diuretics	4706 (51.8)	1455 (53.2)	3251 (51.1)
Thiazide diuretics	668 (7.3)	223 (8.2)	445 (7.0)
Tolvaptan	718 (7.9)	146 (5.3)	572 (9.0)

*SD* standard deviation, *BNP* brain natriuretic peptide, *NT-proBNP* N-terminal pro-brain natriuretic peptide, *ACEi *angiotensin-converting enzyme inhibitor, *ARB* angiotensin-receptor blocker, *MRA* mineralocorticoid receptor antagonist, *SGLT-2i* sodium-glucose cotransporter-2 inhibitor, *HF* heart failure.

*Occurred within one year before the index HF hospitalization.

**Used during the period of 183 days before the index date.

**Table 2:** Characteristics of new users based on different heart failure medication classes.

	ACEi (N = 357)	ARB (N = 304)	MRA (N = 773)	Beta-blockers (N = 695)	SGLT-2i (N = 109)	Digoxin/digitoxin (N = 91)	Loop diuretics (N = 725)	Thiazide diuretics (N = 198)	Tolvaptan (N = 709)
Age (years)									
Mean ± SD	76.5 ± 10.6	76.0 ± 11.1	78.0 ± 9.9	75.7 ± 11.4	71.3 ± 11.6	77.2 ± 9.4	78.1 ± 9.4	78.5 ± 9.0	78.7 ± 9.3
Gender, male, n (%)	212 (59.4)	151 (49.7)	427 (55.2)	370 (53.2)	71 (65.1)	43 (47.3)	368 (50.8)	102 (51.5)	401 (56.6)
BNP (pg/mL)									
Patients with recorded BNP values, n (%)	263 (73.7)	222 (73.0)	561 (72.6)	512 (73.7)	86 (78.9)	76 (83.5)	470 (64.8)	152 (76.8)	539 (76.0)
Mean ± SD	468.0 ± 693.6	413.0 ± 551.6	410.1 ± 512.1	396.3 ± 611.1	386.7 ± 432.0	368.0 ± 342.5	253.2 ± 275.0	371.8 ± 407.9	422.3 ± 492.4
BNP/NT-proBNP category, n (%)									
BNP ≤ 100 or NT-proBNP ≤ 400 pg/mL	48 (13.4)	60 (19.7)	124 (16.0)	157 (22.6)	24 (22.0)	13 (14.3)	181 (25.0)	39 (19.7)	108 (15.2)
100 < BNP ≤ 200 or 400 < NT proBNP ≤ 900 pg/mL	60 (16.8)	47 (15.5)	120 (15.5)	100 (14.4)	18 (16.5)	16 (17.6)	111 (15.3)	25 (12.6)	95 (13.4)
200 < BNP ≤ 300 or 900 < NT proBNP ≤ 2000 pg/mL	36 (10.1)	33 (10.9)	101 (13.1)	80 (11.5)	15 (13.8)	14 (15.4)	67 (9.2)	30 (15.2)	106 (15.0)
BNP > 300 or NT-proBNP ≥ 2000 pg/mL	146 (40.9)	102 (33.6)	276 (35.7)	220 (31.7)	35 (32.1)	38 (41.8)	160 (22.1)	78 (39.4)	284 (40.1)
Missing	67 (18.8)	62 (20.4)	152 (19.7)	138 (19.9)	17 (15.6)	10 (11.0)	206 (28.4)	26 (13.1)	116 (16.4)
Comorbidity, n (%)									
Hypertension	294 (82.4)	254 (83.6)	668 (86.4)	579 (83.3)	98 (89.9)	77 (84.6)	634 (87.4)	181 (91.4)	625 (88.2)
Chronic kidney disease	197 (55.2)	184 (60.5)	430 (55.6)	398 (57.3)	78 (71.6)	62 (68.1)	355 (49.0)	139 (70.2)	523 (73.8)
Ischemic heart disease	199 (55.7)	158 (52.0)	433 (56.0)	384 (55.3)	68 (62.4)	48 (52.7)	385 (53.1)	118 (59.6)	396 (55.9)
Atrial fibrillation	152 (42.6)	118 (38.8)	333 (43.1)	251 (36.1)	43 (39.4)	68 (74.7)	261 (36.0)	77 (38.9)	344 (48.5)
Diabetes mellitus	109 (30.5)	98 (32.2)	253 (32.7)	245 (35.3)	75 (68.8)	28 (30.8)	250 (34.5)	77 (38.9)	257 (36.2)
Stroke	84 (23.5)	60 (19.7)	160 (20.7)	147 (21.2)	26 (23.9)	17 (18.7)	165 (22.8)	37 (18.7)	139 (19.6)
Myocardial infarction	72 (20.2)	49 (16.1)	153 (19.8)	109 (15.7)	24 (22.0)	15 (16.5)	128 (17.7)	30 (15.2)	129 (18.2)
Chronic obstructive pulmonary disease	66 (18.5)	49 (16.1)	153 (19.8)	116 (16.7)	15 (13.8)	20 (22.0)	112 (15.4)	40 (20.2)	148 (20.9)
Anemia	113 (31.7)	105 (34.5)	238 (30.8)	206 (29.6)	16 (14.7)	26 (28.6)	216 (29.8)	64 (32.3)	283 (39.9)
Hyperkalemia	34 (9.5)	32 (10.5)	43 (5.6)	78 (11.2)	10 (9.2)	5 (5.5)	61 (8.4)	36 (18.2)	109 (15.4)
Hypotension	8 (2.2)	6 (2.0)	14 (1.8)	13 (1.9)	1 (0.9)	2 (2.2)	10 (1.4)	3 (1.5)	17 (2.4)
HF treatments before the index date*, n (%)									
ACEi	0 (0)	66 (21.7)	104 (13.5)	73 (10.5)	13 (11.9)	17 (18.7)	91 (12.6)	23 (11.6)	112 (15.8)
ARB	122 (34.2)	0 (0)	298 (38.6)	308 (44.3)	57 (52.3)	30 (33.0)	352 (48.6)	101 (51.0)	294 (41.5)
MRA	72 (20.2)	48 (15.8)	0 (0)	110 (15.8)	23 (21.1)	31 (34.1)	61 (8.4)	40 (20.2)	196 (27.6)
Beta-blockers	152 (42.6)	114 (37.5)	325 (42.0)	0 (0)	54 (49.5)	47 (51.6)	313 (43.2)	79 (39.9)	346 (48.8)
SGLT-2i	10 (2.8)	6 (2.0)	16 (2.1)	19 (2.7)	0 (0)	4 (4.4)	15 (2.1)	6 (3.0)	11 (1.6)
Digoxin/digitoxin	16 (4.5)	9 (3.0)	45 (5.8)	32 (4.6)	4 (3.7)	0 (0)	33 (4.6)	7 (3.5)	41 (5.8)
Loop diuretics	198 (55.5)	178 (58.6)	439 (56.8)	352 (50.6)	69 (63.3)	61 (67.0)	0 (0)	135 (68.2)	532 (75.0)
Thiazide diuretics	25 (7.0)	18 (5.9)	55 (7.1)	57 (8.2)	9 (8.3)	9 (9.9)	72 (9.9)	0 (0)	77 (10.9)
Tolvaptan	26 (7.3)	17 (5.6)	45 (5.8)	28 (4.0)	16 (14.7)	9 (9.9)	16 (2.2)	24 (12.1)	0 (0)

*SD* standard deviation, *BNP* brain natriuretic peptide, *NT-proBNP *N-terminal pro-brain natriuretic peptide, *ACEi* angiotensin-converting enzyme inhibitor, *ARB* angiotensin-receptor blocker, *MRA* mineralocorticoid receptor antagonist, *SGLT-2i* sodium-glucose cotransporter-2 inhibitor, *HF* heart failure.

*Used during the period of 183 days before the index date.

### Drug utilization patterns before and after the index hospitalization for HF

**Figure 3:**
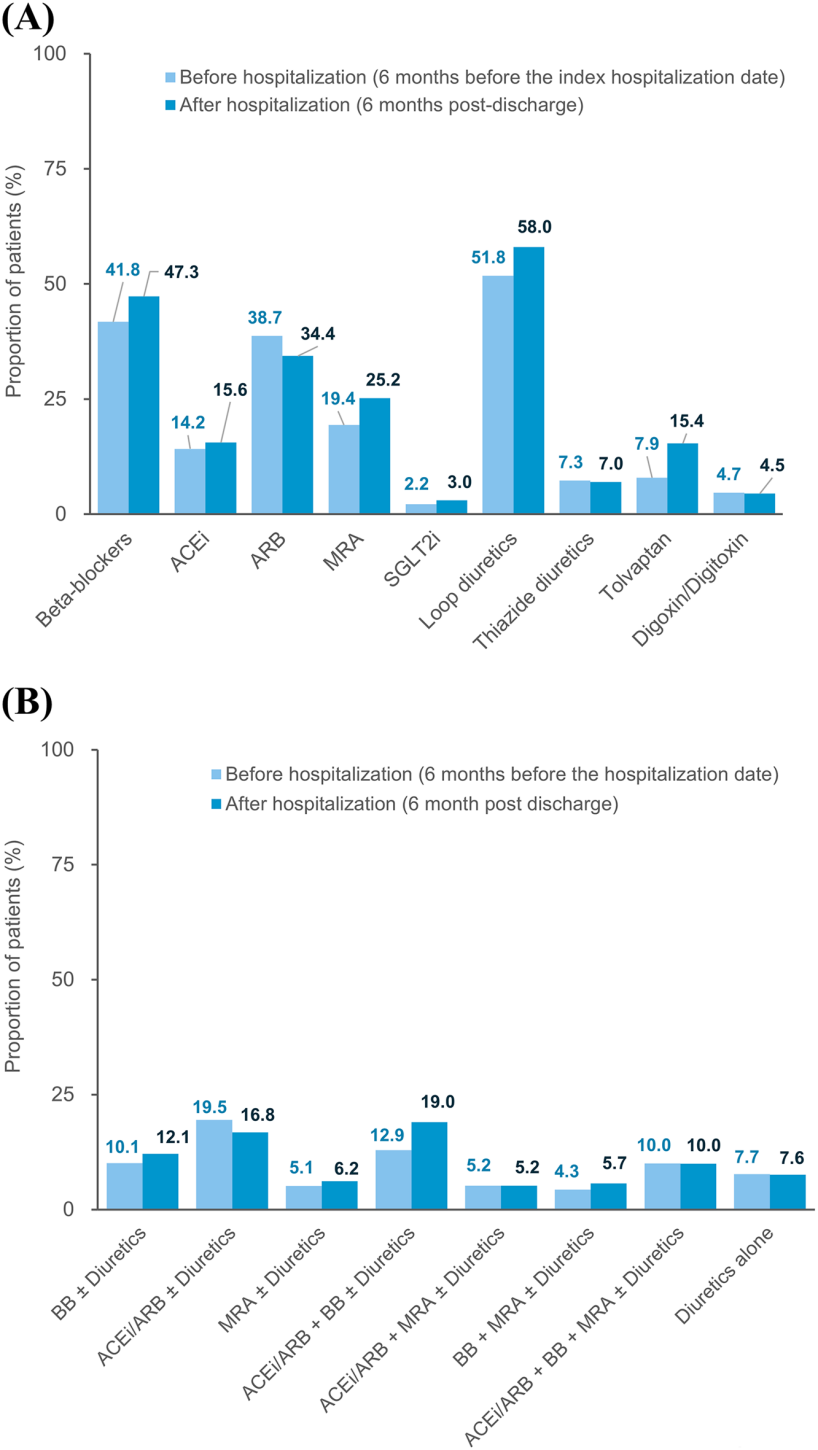
Drug utilization patterns before and after the index hospitalization for heart failure. Panel (**A**) shows the drug utilization patterns for each HF medication class, and panel (**B**) shows the combination patterns of HF medications. The denominator is all patients included in the study (n = 9091). *ACEi* angiotensin-converting-enzyme inhibitor, *ARB* angiotensin receptor blocker, *BB* beta-blockers, *MRA* mineralocorticoid receptor antagonist, *SGLT-2i* sodium-glucose co-transporter-2 inhibitor.

**Figure 4:**
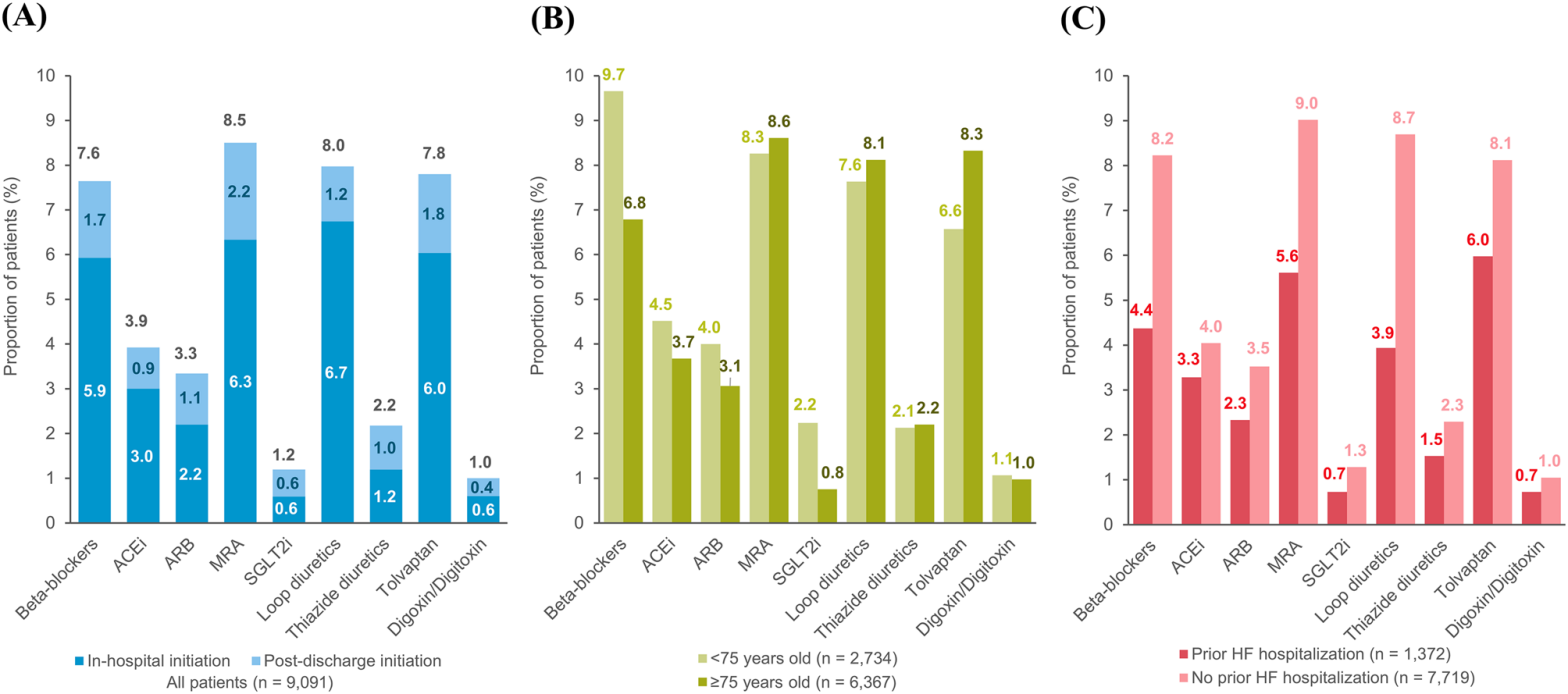
Distribution of newly initiated HF medication classes in all patients and subgroups of patients stratified based on age and a history of prior hospitalization for HF. Panel (**A**) shows the results for the group with all patients (n = 9091), panel (**B**) shows the results for the subgroups of patients with age < 75 years (n = 2724) or ≥ 75 years (n = 6367), and panel (**C**) shows the results for the subgroups of patients with (n = 1372) or without a history of hospitalization for HF before the index hospitalization (n = 7719). *ACEi* angiotensin-converting-enzyme inhibitor, *ARB* angiotensin receptor blocker, *MRA* mineralocorticoid receptor antagonist, *SGLT-2i* sodium-glucose co-transporter-2 inhibitor, *HF* heart failure.

The drug utilization patterns before and after the index hospitalization for HF are shown in Fig. 3. Before the index hospitalization, loop diuretics (51.8%), beta-blockers (41.8%), ARB (38.7%), and MRA (19.4%) were the four most prescribed HF medications. After the index hospitalization, the proportions of patients prescribed with beta-blockers, MRAs, loop diuretics, and tolvaptan increased. Among the combinations of HF medications, the increasing use was most pronounced in the combination of an ACEi/ARB and a beta-blocker (from 12.9 to 19.0%). The distributions of newly initiated HF medications are depicted in Fig. 4. MRAs (8.5%), loop diuretics (8.0%), tolvaptan (7.8%), and beta-blockers (7.6%) were the four most frequently added medication classes after HF hospitalization, which was followed by ACEi/ARB (7.3%). A third (35.1%) of patients were treated with HF monotherapy (beta-blockers, ACEi/ARB, or MRA) and 7.6% by diuretics alone after the index hospitalization. The proportion of new users of beta-blockers was higher in the subgroup aged < 75 years than in the subgroup aged ≥ 75 years. In patients with a history of prior HF hospitalization, proportions of new users of MRA and tolvaptan were higher than those of the other medication classes.

### Adherence and persistence to the newly initiated HF medications

**Figure 5:**
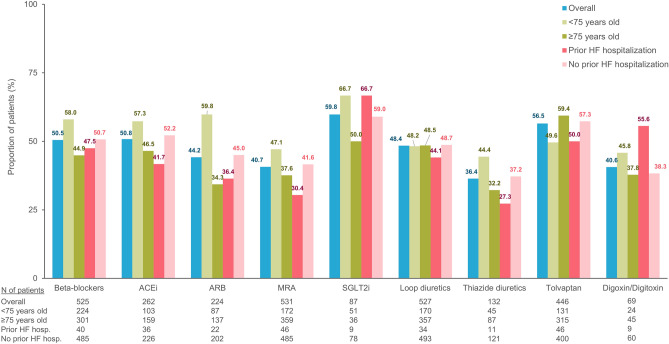
Proportion of patients with continuous use of HF medications during the first year after initiation. The analysis was performed in patients who could be followed up for 365 days after HF treatment initiation in each new-user cohort. *ACEi* angiotensin-converting-enzyme inhibitor, *ARB* angiotensin receptor blocker, *HF* heart failure, *MRA* mineralocorticoid receptor antagonist, *SGLT-2i* sodium-glucose co-transporter-2 inhibitor.

Table 3 shows the PDC of newly initiated HF medications summarized based on different medication classes. The mean ± SD value of PDC of newly started HF medications ranged from 0.57 ± 0.37 in new users of thiazide diuretics to the highest 0.77 ± 0.32 in new users of SGLT-2i. Patients with age ≥ 75 years exhibited consistently lower PDC values than those aged < 75 years for all medication classes except for tolvaptan. Likewise, PDC values in patients with a history of hospitalization for HF were consistently lower than in patients without a prior history of such hospitalization for all medication classes except for digoxin/ digitoxin. The proportion of patients with continuous use of HF medications during the first year after initiation summarized by different classes are shown in Fig. 5. The proportions of patients with one-year continuous treatment episodes were highest in new users of SGLT-2i (59.8%), followed by those of tolvaptan (56.5%), ACEi (50.8%), and beta-blockers (50.5%). The lowest proportions of one-year continuous treatment episodes were observed in new users of thiazide diuretics (36.4%), followed by those of digoxin/ digitoxin (40.6%) and MRA (40.7%). The proportions of patients with one-year continuous HF medication use were lower in patients with age ≥ 75 years or with a history of hospitalization for HF than in patients aged < 75 years or without a prior history of hospitalization for HF, respectively. However, there were exceptions in higher continuation rates of tolvaptan in patients aged ≥ 75 years than in patients aged < 75 years and digoxin/ digitoxin in patients with a history of hospitalization for HF than in patients without a prior history of hospitalization for HF. These findings were consistent for PDC analysis and the assessment of one-year continuous treatment episodes using the 60-day prescription gap to determine the discontinuation episodes (Supplementary Table S6; Supplementary Fig. S2).

**Table 3:** Proportion of days covered in all patients and subgroups, stratified based on age and with a history of prior hospitalization for heart failure.

	Overall	< 75 years	≥ 75 years	With prior HF hospitalization*	No prior HF hospitalization*
Proportion of days covered**, mean ± SD					
ACEi	0.72 ± 0.36	0.77 ± 0.33	0.68 ± 0.37	0.66 ± 0.35	0.73 ± 0.36
ARB	0.66 ± 0.37	0.79 ± 0.33	0.58 ± 0.38	0.66 ± 0.36	0.66 ± 0.37
MRA	0.64 ± 0.37	0.69 ± 0.37	0.61 ± 0.37	0.58 ± 0.38	0.64 ± 0.37
Beta-blockers	0.70 ± 0.37	0.77 ± 0.34	0.64 ± 0.38	0.67 ± 0.37	0.70 ± 0.36
SGLT-2i	0.77 ± 0.32	0.79 ± 0.32	0.74 ± 0.32	0.76 ± 0.36	0.77 ± 0.32
Digoxin/ digitoxin	0.61 ± 0.37	0.65 ± 0.36	0.60 ± 0.37	0.71 ± 0.35	0.60 ± 0.37
Loop diuretics	0.69 ± 0.36	0.70 ± 0.35	0.69 ± 0.36	0.65 ± 0.37	0.69 ± 0.36
Thiazide diuretics	0.57 ± 0.37	0.63 ± 0.37	0.55 ± 0.37	0.51 ± 0.38	0.58 ± 0.37
Tolvaptan	0.74 ± 0.35	0.71 ± 0.35	0.76 ± 0.35	0.65 ± 0.39	0.75 ± 0.35

*SD* standard deviation, *ACEi* angiotensin-converting enzyme inhibitor, *ARB* angiotensin-receptor blocker, *MRA* mineralocorticoid receptor antagonist, *SGLT-2i* sodium-glucose cotransporter-2 inhibitor, *HF* heart failure. *Hospitalization for HF occurred within 12 months before the index hospitalization. **The analyses were performed in patients who could be followed up for 365 days after the initiation of HF treatment in each new-user cohort. The treatment discontinuation was assessed based on the absence of a continuous prescription record of the HF treatment of interest for 30 days.

## Discussion

In this real-world study, we present drug utilization patterns before and after worsening HF events, including the initiation of HF medications and persistence was measured as continuous treatment episodes across the year following HF treatment initiation.

In the study population, 70% of patients were with age ≥ 75 years, characterizing the large proportion of elderly population of HF patients in real-world settings as recently reported^22^. Patients were frequently comorbid with hypertension, CKD, ischemic heart disease, and atrial fibrillation. These findings were in line with the recent reports from large-scale registries in Japan^9,20^. We found that one-third of patients initiated new HF medications, of which roughly three in four patients started these medications during hospitalization for HF. After the hospitalization, > 40% of patients were treated with monotherapy of the foundational HF therapy (The term "foundational HF therapy" is used instead of "GDMT" since SGLT2i and ARNI are not included here) or diuretics alone. The drug utilization patterns before the index hospitalizations were similar to those reported by JROADHF^9^, with diuretics (55.9% in this study and 54.9% in JROADHF), ACEi/ARB (51.8% vs. 46.2%), and beta-blockers (41.8% vs. 36.3%) as the three most prescribed medication classes. However, the drug utilization rates after the index hospitalizations were lower than those reported by JROADHF for several foundational medications, such as beta-blockers (47.3% in this study and 63.7% in JROADHF), ACEis (15.6% vs. 30.1%), and MRAs (25.2% vs. 50.9%). As the institutions participating in JROADHF were mainly Japanese Circulation Society-certified teaching hospitals, requiring board-certified cardiologists and cardiovascular beds^9^, these differences can partially be attributed to the differences in the facilities covered in the present study. Despite the evidence of HF diagnosis and treatment before the index hospitalization, 40% of patients did not explicitly have a record of a cardiologist visit. Given the non-selective inclusion of care providers, our study population and inherent findings should benefit from higher generalizability than prior evidence. The study results show variability across implementing guideline recommended therapies^30^ by different facilities as reported in the previous study^31^.

The initiated HF medication classes were divided mainly into foundational medications (beta-blockers, ACEi/ARB, or MRAs) and treatment to alleviate congestion (loop diuretics or tolvaptan). A few differences in the treatment initiation patterns were observed in patients aged ≥ 75 years, including low initiation rates of beta-blockers, suggesting concerns regarding tolerability in older patients^32^. Reasons for not using GDMT may be multifold; however, the findings from this study overall reflect the challenges related to the substantial underuse of GDMT. A recent review reported that renal dysfunction, hypotension, and hyperkalemia were the common reasons for not prescribing ACEi/ARB and MRA, whereas those for not prescribing beta-blockers were bradycardia, hypotension, and asthma/ chronic obstructive pulmonary disease^33^. These conditions were prevalent in older patients (i.e. aged ≥ 75 years) and patients with a history of hospitalization for HF, suggesting the importance of early initiation of GDMT before reaching the deteriorating conditions with repeated HF hospitalizations. Clinical inertia, i.e., continuing the existing treatment rather than optimizing, is also discussed as a reason for not achieving the target treatment regimen^34^.

This study also highlighted the low adherence to the HF medications added after the worsening HF event. The mean PDC of all HF medication classes was < 0.80, i.e. below the commonly used threshold for adherence^35^. High discontinuation rates of newly initiated HF medications were also found in recent multinational observational studies in the United States, the United Kingdom, Sweden, and Japan^21,36,37^. The low adherence to HF medications was relatively more pronounced in older patients and in patients with a history of hospitalization for HF, which is an important clinical issue, as these patient populations account for a large portion of post-discharge mortality and re-hospitalization for HF^38–40^. A recent meta-analysis suggested that the combination of ARNI, beta-blockers, MRAs, and SGLT-2i effectively reduced HF-associated mortality, with 5 life-years gained for a 70-year-old patient^41^. In a multinational STRONG-HF trial, an intensive treatment strategy of the up-titration of GDMT and close follow-up after an acute HF admission was shown to be tolerable with reduced risks of all-cause death or HF readmission and improved quality of life^42^. However, these studies focused on relatively younger populations compared with patients in real-world clinical settings; therefore, data on the tolerability and up-titration of GDMT in specific population groups, e.g., older HF patients, is required. A relatively more individualized approach using digital therapeutics to support treatment continuations has also been tested in a pilot study^43^. This type of intervention has already been proven effective in improving the quality of life and prolonging survival in other diseases through the early detection of adverse events associated with the treatment and disease progression^44^. An integrated approach in HF care results in a relatively more individualized and optimized treatment for HF^45^, which should also provide beneficial effects by supporting continuous use of HF medications for those vulnerable patients for treatment discontinuation.

This study has several limitations. First, the dataset did not cover the period after the authorization of ivabradine and ARNI in Japan. Furthermore, SGLT-2i was indicated for type 2 diabetes but not for HF (approved in November 2020) during the period when drug utilization patterns were assessed. Therefore, information regarding these relatively new HF drugs was limited. Second, echocardiography data was unavailable in the dataset, precluding left ventricular ejection fraction analyses. Therefore, we could not categorize patients based on the ejection fraction, i.e., HF with reduced, mildly reduced, or preserved ejection fraction, in the analysis. Third, RWD collected only structured information. Therefore, qualitative information could not be assessed, such as reasons for treatment initiation/ discontinuation. Notably, patients could not be followed up across different hospitals in RWD, which could have resulted in limited follow-up observability for those patients.

In this study, we present comprehensive information on HF drug utilization patterns in a representative sample of real-world HF patients. Despite the proven efficacy of pharmacological therapies for HF, only one-third of patients had received the newly initiated HF medications and less than half of patients were treated with foundational monotherapy or diuretics alone after worsening HF events, suggesting the suboptimal use of these medications. The consistently low adherence of newly started HF medications post-hospitalization for HF, despite the high incidence of all-cause death and HF readmission, underscores the importance of continuous efforts for treatment maintenance while highlighting older patients and patients with a history of hospitalization for HF as relatively more vulnerable groups for treatment discontinuations. These findings should provide important insights in real-world treatment patterns after worsening HF events as critical aspects to optimize the beneficial effects of pharmacological therapies for HF. They suggest the need of additional options to optimize treatment in order to lower residual risks in HF patients. It remains to be studied how newly available treatment options (e.g., ARNI and SGLT-2is) as well as drugs which have specifically been investigated in patients with worsening HF (vericiguat) lead to improved outcomes in real world settings.

## Supplementary Information

Supplementary Information.

## Data Availability

All necessary data required to interpret and conclude the findings of this study were included in the main text and supplementary materials.
