# Author Correction: Initiation and continuation of pharmacological therapies in patients hospitalized for heart failure in Japan

**DOI:** 10.1038/s41598-024-75133-6

**Published:** 2024-10-15

**Authors:** Suguru Okami, Coralie Lecomte, Hanaya Raad, Mireia Aguila, Zuzana Mohrova, Makiko Takeichi, Takanori Tsuchiya, Christoph Ohlmeier, Thomas Evers, Alexander Michel

**Affiliations:** 1 Medical Affairs & Pharmacovigilance, Bayer Yakuhin Ltd, 2-4-9 Umeda, Kita-Ku, Osaka, Breeze Tower, 530-0001 Japan; 2 Aetion Inc, 5 Penn Plaza, New York, USA; 3 Market Access & Public Affairs, Bayer Yakuhin, Ltd, 2-4-9 Umeda, Kita-Ku, Osaka, Japan; 4 Integrated Evidence Generation & Business Innovation, Bayer AG, 13342 Berlin, Germany; 5 Integrated Evidence Generation & Business Innovation, Bayer AG, 42096 Wuppertal, Germany; 6 Integrated Evidence Generation & Business Innovation, Bayer Consumer Care AG, Peter Merian Straße 84, 4052 Basel, Switzerland

Correction to: *Scientific Reports *10.1038/s41598-024-60011-y, published online 20 April 2024

The original version of this Article contained errors in Tables 1 and 2 where the number and percentage of comorbidity reported were incorrect.

**Table 1:** Baseline characteristics in all patients, including new and non-new users of heart failure treatment. *SD* standard deviation, *BNP* brain natriuretic peptide, *NT-proBNP* N-terminal pro-brain natriuretic peptide, *ACEi* angiotensin-converting enzyme inhibitor, *ARB* angiotensin-receptor blocker, *MRA* mineralocorticoid receptor antagonist, *SGLT-2i* sodium-glucose cotransporter-2 inhibitor, *HF* heart failure. *Occurred within one year before the index HF hospitalization. **Used during the period of 183 days before the index date.

	**Overall** **(N = 9091)**	**New users** **(N = 2735)**	**Non-new users (N = 6356)**
Age (years)			
Mean ± SD	77.6 ± 10.0	77.4 ± 10.3	77.6 ± 9.9
Age group, n (%)			
< 40	56 (0.6)	19 (0.7)	37 (0.6)
40–64	841 (9.3)	253 (9.3)	588 (9.3)
65–74	1827 (20.1)	561 (20.5)	1266 (19.9)
≥ 75	6367 (70.0)	1902 (69.5)	4465 (70.2)
Gender, male, n (%)	5007 (55.1)	1477 (54.0)	3530 (55.5)
BNP (pg/mL)			
Patients with recorded BNP values, n (%)	6534 (71.9)	2008 (73.4)	4526 (71.2)
Mean ± SD	336.6 ± 509.9	374.9 ± 499.3	319.7 ± 513.7
BNP/NT-proBNP category, n (%)			
BNP ≤ 100 or NT-proBNP ≤ 400 pg/mL	2302 (25.3)	541 (19.8)	1761 (27.7)
100 < BNP ≤ 200 or 400 < NT-proBNP ≤ 900 pg/mL	1366 (15.0)	413 (15.1)	953 (15.0)
200 < BNP ≤ 300 or 900 < NT-proBNP ≤ 2000 pg/mL	991 (10.9)	340 (12.4)	651 (10.2)
BNP > 300 or NT-proBNP ≥ 2000 pg/mL	2548 (28.0)	913 (33.4)	1635 (25.7)
Missing	1884 (20.7)	528 (19.3)	1356 (21.3)
Comorbidity, n (%)			
Hypertension	5464 (60.1)	1540 (56.3)	3924 (61.7)
Chronic kidney disease	5005 (55.1)	1489 (54.4)	3516 (55.3)
Ischemic heart disease	3721 (40.9)	1044 (38.2)	2677 (42.1)
Atrial fibrillation	2584 (28.4)	779 (28.5)	1805 (28.4)
Diabetes mellitus	2352 (25.9)	656 (24.0)	1696 (26.7)
Stroke	1323 (14.6)	387 (14.1)	936 (14.7)
Myocardial infarction	1301 (14.3)	319 (11.7)	982 (15.4)
Chronic obstructive pulmonary disease	1096 (12.1)	297 (10.9)	799 (12.6)
Anemia	2145 (23.6)	576 (21.1)	1569 (24.7)
Hyperkalemia	692 (7.6)	199 (7.3)	493 (7.8)
Hypotension	244 (2.7)	38 (1.4)	206 (3.2)
Cardiovascular procedure, n (%)			
Cardiac resynchronization therapy	215 (2.4)	65 (2.4)	150 (2.4)
Implantable cardioverter defibrillator	41 (0.5)	16 (0.6)	25 (0.4)
Having a history of prior hospitalization for HF*, n (%)	1372 (15.1)	290 (10.6)	1082 (17.0)
HF treatments before the index date**, n (%)			
ACEi	1291 (14.2)	376 (13.7)	915 (14.4)
ARB	3519 (38.7)	1075 (39.3)	2444 (38.5)
MRA	1765 (19.4)	461 (16.9)	1304 (20.5)
Beta-blockers	3801 (41.8)	1058 (38.7)	2743 (43.2)
SGLT-2i	198 (2.2)	59 (2.2)	139 (2.2)
Digoxin/digitoxin	424 (4.7)	141 (5.2)	283 (4.5)
Loop diuretics	4706 (51.8)	1455 (53.2)	3251 (51.1)
Thiazide diuretics	668 (7.3)	223 (8.2)	445 (7.0)
Tolvaptan	718 (7.9)	146 (5.3)	572 (9.0)

**Table 2:** Characteristics of new users based on different heart failure medication classes. *SD* standard deviation, *BNP* brain natriuretic peptide, *NT-proBNP* N-terminal pro-brain natriuretic peptide, *ACEi* angiotensin-converting enzyme inhibitor, *ARB* angiotensin-receptor blocker, *MRA* mineralocorticoid receptor antagonist, *SGLT-2i* sodium-glucose cotransporter-2 inhibitor, *HF* heart failure. *Used during the period of 183 days before the index date.

	**ACEi** **(N = 357)**	**ARB** **(N = 304)**	**MRA** **(N = 773)**	**Beta-blockers (N = 695)**	**SGLT-2i** **(N = 109)**	**Digoxin/digitoxin (N = 91)**	**Loop diuretics (N = 725)**	**Thiazide diuretics (N = 198)**	**Tolvaptan** **(N = 709)**
Age (years)									
Mean ± SD	76.5 ± 10.6	76.0 ± 11.1	78.0 ± 9.9	75.7 ± 11.4	71.3 ± 11.6	77.2 ± 9.4	78.1 ± 9.4	78.5 ± 9.0	78.7 ± 9.3
Gender, male, n (%)	212 (59.4)	151 (49.7)	427 (55.2)	370 (53.2)	71 (65.1)	43 (47.3)	368 (50.8)	102 (51.5)	401 (56.6)
BNP (pg/mL)									
Patients with recorded BNP values, n (%)	263 (73.7)	222 (73.0)	561 (72.6)	512 (73.7)	86 (78.9)	76 (83.5)	470 (64.8)	152 (76.8)	539 (76.0)
Mean ± SD	468.0 ± 693.6	413.0 ± 551.6	410.1 ± 512.1	396.3 ± 611.1	386.7 ± 432.0	368.0 ± 342.5	253.2 ± 275.0	371.8 ± 407.9	422.3 ± 492.4
BNP/NT-proBNP category, n (%)									
BNP ≤ 100 or NT-proBNP ≤ 400 pg/mL	48 (13.4)	60 (19.7)	124 (16.0)	157 (22.6)	24 (22.0)	13 (14.3)	181 (25.0)	39 (19.7)	108 (15.2)
100 < BNP ≤ 200 or 400 < NT proBNP ≤ 900 pg/mL	60 (16.8)	47 (15.5)	120 (15.5)	100 (14.4)	18 (16.5)	16 (17.6)	111 (15.3)	25 (12.6)	95 (13.4)
200 < BNP ≤ 300 or 900 < NT proBNP ≤ 2000 pg/mL	36 (10.1)	33 (10.9)	101 (13.1)	80 (11.5)	15 (13.8)	14 (15.4)	67 (9.2)	30 (15.2)	106 (15.0)
BNP > 300 or NT-proBNP ≥ 2000 pg/mL	146 (40.9)	102 (33.6)	276 (35.7)	220 (31.7)	35 (32.1)	38 (41.8)	160 (22.1)	78 (39.4)	284 (40.1)
Missing	67 (18.8)	62 (20.4)	152 (19.7)	138 (19.9)	17 (15.6)	10 (11.0)	206 (28.4)	26 (13.1)	116 (16.4)
Comorbidity, n (%)									
Hypertension	199 (55.7)	180 (59.2)	422 (54.6)	363 (52.2)	56 (51.4)	56 (61.5)	395 (54.5)	119 (60.1)	399 (56.3)
Chronic kidney disease	168 (47.1)	163 (53.6)	360 (46.6)	359 (55.5)	69 (63.3)	54 (59.3)	308 (42.5)	119 (60.1)	467 (65.9)
Ischemic heart disease	144 (40.3)	113 (37.2)	295 (38.2)	264 (38.0)	47 (43.1)	33 (36.3)	250 (34.5)	77 (38.9)	243 (34.3)
Atrial fibrillation	115 (32.2)	82 (27.0)	238 (30.8)	157 (22.6)	23 (21.1)	54 (59.3)	166 (22.9)	45 (22.7)	227 (32.0)
Diabetes mellitus	73 (20.4)	63 (20.7)	172 (22.3)	171 (24.6)	50 (45.9)	22 (24.2)	175 (24.1)	54 (27.3)	166 (23.4)
Stroke	57 (16.0)	42 (13.8)	101 (13.1)	93 (13.4)	10 (9.2)	13 (14.3)	119 (16.4)	21 (10.6)	89 (12.6)
Myocardial infarction	54 (15.1)	33 (10.9)	98 (12.7)	66 (9.5)	14 (12.8)	12 (13.2)	78 (10.8)	18 (9.1)	79 (11.1)
Chronic obstructive pulmonary disease	43 (12.0)	28 (9.2)	86 (11.1)	63 (9.1)	5 (4.6)	9 (9.9)	66 (9.1)	20 (10.1)	83 (11.7)
Anemia	76 (21.3)	68 (22.4)	152 (19.7)	117 (16.8)	8 (7.3)	16 (17.6)	121 (16.7)	40 (20.2)	182 (25.7)
Hyperkalemia	24 (6.7)	22 (7.2)	31 (4.0)	50 (7.2)	4 (3.7)	4 (4.4)	36 (5.0)	25 (12.6)	70 (9.9)
Hypotension	5 (1.4)	4 (1.3)	10 (1.3)	12 (1.7)	0 (0)	1 (1.1)	6 (0.8)	2 (1.0)	13 (1.8)
HF treatments before the index date*, n (%)									
ACEi	0 (0)	66 (21.7)	104 (13.5)	73 (10.5)	13 (11.9)	17 (18.7)	91 (12.6)	23 (11.6)	112 (15.8)
ARB	122 (34.2)	0 (0)	298 (38.6)	308 (44.3)	57 (52.3)	30 (33.0)	352 (48.6)	101 (51.0)	294 (41.5)
MRA	72 (20.2)	48 (15.8)	0 (0)	110 (15.8)	23 (21.1)	31 (34.1)	61 (8.4)	40 (20.2)	196 (27.6)
Beta-blockers	152 (42.6)	114 (37.5)	325 (42.0)	0 (0)	54 (49.5)	47 (51.6)	313 (43.2)	79 (39.9)	346 (48.8)
SGLT-2i	10 (2.8)	6 (2.0)	16 (2.1)	19 (2.7)	0 (0)	4 (4.4)	15 (2.1)	6 (3.0)	11 (1.6)
Digoxin/digitoxin	16 (4.5)	9 (3.0)	45 (5.8)	32 (4.6)	4 (3.7)	0 (0)	33 (4.6)	7 (3.5)	41 (5.8)
Loop diuretics	198 (55.5)	178 (58.6)	439 (56.8)	352 (50.6)	69 (63.3)	61 (67.0)	0 (0)	135 (68.2)	532 (75.0)
Thiazide diuretics	25 (7.0)	18 (5.9)	55 (7.1)	57 (8.2)	9 (8.3)	9 (9.9)	72 (9.9)	0 (0)	77 (10.9)
Tolvaptan	26 (7.3)	17 (5.6)	45 (5.8)	28 (4.0)	16 (14.7)	9 (9.9)	16 (2.2)	24 (12.1)	0 (0)

Additionally, Supplementary Information Table S5 contained the same error. The original Table S5 and accompanying legend appear below.

**Table S5:** Characteristics of new users of heart failure treatment in subgroups stratified based on age and a history of prior hospitalization for heart failure.

	**<75 years old** **(N = 833)**	**≥75 years old** **(N = 1,902)**	With prior HF hospitalization* (N = 290)	No prior HF hospitalization* (N =2,445)
Age (years)				
Mean ± SD	64.9 ± 9.3	82.9 ± 3.8	79.1 ± 9.4	77.2 ± 10.3
Gender, male, n (%)	567 (68.1)	910 (47.8)	146 (50.3)	1,331 (54.4)
BNP (pg/mL)				
Patients with recorded BNP values, n (%)	627 (75.3)	1,381 (72.6)	233 (80.3)	1,775 (72.6)
Mean ± SD	359.9 ± 556.8	381.6 ± 470.9	475.5 ± 481.5	361.7 ± 500.2
BNP/NT-proBNP category, n (%)				
BNP ≤100 or NT-proBNP ≤400 pg/mL	247 (29.7)	294 (15.5)	40 (13.8)	501 (20.5)
100< BNP ≤200 or 400< NT-proBNP ≤900 pg/mL	100 (12.0)	313 (16.5)	42 (14.5)	371 (15.2)
200< BNP ≤300 or 900< NT-proBNP ≤2000 pg/mL	79 (9.5)	261 (13.7)	44 (15.2)	296 (12.1)
BNP >300 or NT-proBNP ≥2000 pg/mL	240 (28.8)	673 (35.4)	134 (46.2)	779 (31.9)
Missing	167 (20.0)	361 (19.0)	30 (10.3)	498 (20.4)
Comorbidity, n (%)				
Hypertension	451 (54.1)	1,089 (57.3)	221 (76.2)	1,319 (53.9)
Chronic kidney disease	403 (48.3)	1,086 (57.1)	151 (52.1)	1,338 (46.5)
Ischemic heart disease	339 (40.7)	705 (37.1)	150 (51.7)	894 (36.6)
Atrial fibrillation	197 (23.6)	582 (30.6)	121 (41.7)	658 (26.9)
Diabetes mellitus	232 (27.9)	424 (22.3)	91 (31.4)	565 (23.1)
Stroke	93 (11.2)	294 (15.5)	58 (20.0)	329 (13.5)
Myocardial infarction	119 (14.3)	200 (10.5)	56 (19.3)	263 (10.8)
Chronic obstructive pulmonary disease	69 (8.3)	228 (12.0)	50 (17.2)	247 (10.1)
Anemia	154 (18.5)	422 (22.2)	108 (37.2)	468 (19.1)
Hyperkalemia	57 (6.8)	142 (7.5)	22 (7.6)	177 (7.2)
Hypotension	10 (1.2)	28 (1.5)	10 (3.4)	28 (1.1)
HF treatments prior to the index date,** n (%)				
ACEi	135 (16.2)	241 (12.7)	49 (16.9)	327 (13.4)
ARB	347 (41.7)	728 (38.3)	65 (22.4)	1,010 (41.3)
MRA	157 (18.8)	304 (16.0)	50 (17.2)	411 (16.8)
Beta-blockers	358 (43.0)	700 (36.8)	118 (40.7)	940 (38.4)
SGLT-2i	25 (3.0)	34 (1.8)	6 (2.1)	53 (2.2)
Digoxin/ digitoxin	43 (5.2)	98 (5.2)	8 (2.8)	133 (5.4)
Loop diuretics	406 (48.7)	1,049 (55.2)	155 (53.4)	1,300 (53.2)
Thiazide diuretics	57 (6.8)	166 (8.7)	16 (5.5)	207 (8.5)
Tolvaptan	49 (5.9)	97 (5.1)	18 (6.2)	128 (5.2)

SD, standard deviation; BNP, brain natriuretic peptide; NT-proBNP, N-terminal pro-brain natriuretic peptide; ACEi, angiotensin-converting enzyme inhibitor; ARB, angiotensin-receptor blocker; MRA, mineralocorticoid receptor antagonist; SGLT-2i, sodium-glucose cotransporter-2 inhibitor; HF, heart failure.

* Hospitalization for HF occurred within 12 months before the index date of hospitalization. ** Used during the period of 183 days before the index date.

Finally, in the Results section, under the subheading ‘Patients characteristics’,

“Further, 60.1, 55.1, 40.9, and 28.4% of patients had hypertension, chronic kidney disease (CKD), ischemic heart disease, and atrial fibrillation, respectively.”

now reads:

“Further, 86.2, 55.9, 56.9, and 39.3% of patients had hypertension, chronic kidney disease (CKD), ischemic heart disease, and atrial fibrillation, respectively.”

The original Article and accompanying Supplementary Information files have been corrected.

