# Supplementary material for: Initiation and continuation of pharmacological therapies in patients hospitalized for heart failure in Japan
Source: Sci Rep. 2024 Apr 20;14:9095. doi: 10.1038/s41598-024-60011-y (PMC11032365; doi:10.1038/s41598-024-60011-y)
Supplement: Supplementary file 1 — Supplementary Information. [file 41598_2024_60011_MOESM1_ESM.pdf]

APPENDIX

Table of contents 1
Supplementary tables and figures 2
Table S1. List of variables used to assess eligibility, comorbidities, and clinical events in the study populations...2
Table S2. List of heart failure treatments assessed in the study 3
Table S3. STROBE Checklist 4
Table S4. Patient characteristics at index heart failure hospitalization...6
Table S5. Characteristics of new users of heart failure treatment in subgroups stratified by age and history of prior heart failure hospitalization..7
Table S6. Sensitivity analysis of proportions of days covered in overall patients and in subgroups stratified by age and history of prior heart failure hospitalization assessed based on the 60-day gaps of prescriptions for discontinuation episodes..8
Figure S1. Kaplan-Meier curves of all-cause death and heart failure hospitalization.......................9
Figure S2. Sensitivity analyses of continuous use of HF medications during the first year after initiation based on the 60-day gap of prescriptions for treatment discontinuation episodes...........10
References..11

Supplementary tables and figures

Table S1. List of variables used to assess eligibility, comorbidities, and clinical events in the study populations.

Variables	Definition
Hypertension	Any inpatient or outpatient record with a diagnosis (any position) of hypertension (ICD-10: I10, I15)
Chronic kidney disease	Any inpatient or outpatient diagnosis (any position) of chronic kidney disease or two eGFR values <60 mL/min/1.73m^2 at least three months apart within a 12-month baseline period from electronic medical records [1].
Ischemic heart disease	Any inpatient or outpatient record with a diagnosis (any position) of myocardial infarction and other ischemic heart diseases (ICD-10: I20, I21, I22)
Atrial fibrillation	Any inpatient or outpatient record with a diagnosis (any position) of atrial fibrillation (ICD-10: I48.0, I48.1, I48.2, I48.9)
Diabetes mellitus	Any inpatient or outpatient record with a diagnosis (any position) of diabetes mellitus (ICD-10: E10, E11, E14)
Stroke	Any inpatient or outpatient record with a diagnosis (any position) of ischemic stroke or intracerebral hemorrhage (ICD-10: I60–68)
Myocardial infarction	Any inpatient or outpatient record with a diagnosis (any position) of myocardial infarction (ICD-10: I21, I22)
COPD	Any inpatient or outpatient record with a diagnosis (any position) of COPD (ICD-10: J44.9)
Anemia	Any inpatient or outpatient record with a diagnosis (any position) of anemia (ICD-10: D50–53, D55, D56, D58, D59, D61, D62, D64)
Hyperkalemia	Any inpatient or outpatient record with a diagnosis (any position) of hyperkalemia (ICD-10: E87.5)
Hypotension	Any inpatient or outpatient record with a diagnosis (any position) of hypokalemia (ICD-10: I95)
All-cause death	Any inpatient and outpatient death records identified in the medical record
HF-related hospitalization	Patients with one main or definite diagnosis in electronic medical records for any HF (ICD-10: I50.x, I11.0) during the hospitalization record with a duration of >1 day

ICD-10, international classification of disease, 10th revision; eGFR, estimated glomerular filtration rate; HF, heart failure; COPD, chronic obstructive pulmonary disease.

Table S2. List of heart failure treatments assessed in the study.

Treatment	Definition (ATC code)
ACEi	C09A, C09B
ARB	C09C, C09DA, C09DB
MRA	C03DA
Beta-blocker	C07
SGLT-2i	A10BK
Digoxin/Digitoxin	C01AA
Loop diuretics	C03C
Thiazide diuretics	C03A
Tolvaptan	C03XA01

ACEi, angiotensin-converting-enzyme inhibitor; ARB, angiotensin receptor blocker; MRA, mineralocorticoid receptor antagonist; SGLT-2i, sodium-glucose cotransporter-2 inhibitor; ATC, anatomical therapeutic chemical classification system.

Table S3. STROBE (Strengthening the Reporting of Observational Studies in Epidemiology) Checklist [2].

	Item No.	Recommendation	Page No.
**Title and abstract**	1	(*a*) Indicate the study design with a commonly used term in the title or abstract	2
		(*b*) Provide in the abstract an informative and balanced summary of what was done and what was found	2
**Introduction**			
Background/ rationale	2	Explain the scientific background and rationale for the investigation being reported	3
Objectives	3	State the specific objectives, including any prespecified hypotheses	4
**Methods**			
Study design	4	Present key elements of study design early in the paper	4
Setting	5	Describe the setting, locations, and relevant dates, including periods of recruitment, exposure, follow-up, and data collection	4
Participants	6	(*a*) *Cohort study*—Specify the eligibility criteria and the sources and methods of selection of participants. Describe methods of follow-up	4
		(*b*) For matched studies, provide matching criteria and the number of exposed and unexposed	N/A
Variables	7	Clearly define all outcomes, exposures, predictors, potential confounders, and effect modifiers. Give diagnostic criteria, if applicable	5
Data sources/ measurement	8	For each variable of interest, give sources of data and details of methods of assessment (measurement). Describe the comparability of assessment methods if there is more than one group	5
Bias	9	Describe any efforts to address potential sources of bias	5
Study size	10	Explain how the study size was arrived at	6
Quantitative variables	11	Explain how quantitative variables were handled in the analyses. If applicable, describe which groupings were chosen and why	6
Statistical methods	12	(*a*) Describe all statistical methods, including those used to control for confounding	5–6
		(*b*) Describe any methods used to examine subgroups and interactions	5–6
		(*c*) Explain how missing data were addressed	5
		(*d*) If applicable, explain how the loss to follow-up was addressed	N/A
		(e) Describe any sensitivity analyses	5

Continued on the next page

**Results**			
Participants	13	(a) Report numbers of individuals at each stage of study—e.g., numbers potentially eligible, examined for eligibility, confirmed eligible, included in the study, completing follow-up, and analyzed	6
		(b) Give reasons for non-participation at each stage	6
		(c) Consider the use of a flow diagram	6
Descriptive data	14	(a) Give characteristics of study participants (e.g., demographic, clinical, social) and information on exposures and potential confounders	7
		(b) Indicate the number of participants with missing data for each variable of interest	8
		(c) Summarise follow-up time (e.g., average and total amount)	6
Outcome data	15	Report numbers of outcome events or summary measures over time	6
Main results	16	(*a*) Give unadjusted estimates and, if applicable, confounder-adjusted estimates and their precision (e.g., 95% confidence interval). Make clear which confounders were adjusted for and why they were included	8
		(*b*) Report category boundaries when continuous variables were categorized	8
		(*c*) If relevant, consider translating estimates of relative risk into absolute risk for a meaningful time period	N/A
Other analyses	17	Report other analyses performed—e.g., analyses of subgroups and interactions, and sensitivity analyses	7, 13
**Discussion**			
Key results	18	Summarize key results with reference to study objectives	15
Limitations	19	Discuss the limitations of the study, taking into account sources of potential bias or imprecision. Discuss both the direction and magnitude of any potential bias	17
Interpretation	20	Give a cautious overall interpretation of results considering objectives, limitations, the multiplicity of analyses, results from similar studies, and other relevant evidence	15
Generalizability	21	Discuss the generalizability (external validity) of the study results	15
**Other information**			
Funding	22	Give the source of funding and the role of the funders for the present study and, if applicable, for the original study on which the present article is based	18

Table S4. Patient characteristics during the index hospitalization for heart failure.

	**Overall patients (N = 9,091)**
Duration of index hospitalization (days)	
mean ± SD	28.3 ± 32.6
median (IQR)	18 (10–34)
Underwent cardiac rehabilitation, n (%)	2,313 (25.4)
First recorded laboratory data after admission	
Hemoglobin (g/dL)	
Patients with recorded hemoglobin value, n (%)	8,616 (94.8)
mean ± SD	11.3 ± 2.3
Albumin (g/dL)	
Patients with recorded albumin value, n (%9	8,180 (90.0)
mean ± SD	3.4 ± 0.6
Sodium (mmol/L)	
Patients with recorded sodium values, n (%)	8,649 (95.1)
mean ± SD	138.7 ± 4.7
Potassium (mmol/L)	
Patients with recorded potassium values, n (%)	8,697 (95.7)
mean ± SD	4.2 ± 0.7
BNP (pg/mL)	
Patients with recorded BNP values, n (%)	6,133 (67.5)
mean ± SD	485.9 ± 726.7
BNP/NT-proBNP category, n (%)	
BNP ≤100 or NT-proBNP ≤400 pg/mL	1,776 (19.5)
100< BNP ≤200 or 400< NT-proBNP ≤900 pg/mL	1,094 (12.0)
200< BNP ≤300 or 900< NT-proBNP ≤2000 pg/mL	807 (8.9)
BNP >300 or NT-proBNP ≥2000 pg/mL	3,129 (34.4)
Missing	2,285 (25.1)

SD, standard deviation; IQR, inter-quartile range; BNP, brain natriuretic peptide; NT-proBNP, N-terminal pro-brain natriuretic peptide.

Table S5. Characteristics of new users of heart failure treatment in subgroups stratified based on age and a history of prior hospitalization for heart failure.

	**<75 years old (N = 833)**	**≥75 years old (N = 1,902)**	**With prior HF hospitalization* (N = 290)**	**No prior HF hospitalization* (N =2,445)**
Age (years)				
mean ± SD	64.9 ± 9.3	83.0 ± 3.8	79.2 ± 9.4	77.3 ± 10.3
Gender, male, n (%)	567 (68.1)	910 (47.8)	146 (50.3)	1,331 (54.4)
BNP (pg/mL)				
Patients with recorded BNP values, n (%)	627 (75.3)	1,381 (72.6)	233 (80.3)	1,775 (72.6)
mean ± SD	359.9 ± 556.8	381.6 ± 470.9	475.5 ± 481.5	361.7 ± 500.2
BNP/NT-proBNP category, n (%)				
BNP ≤100 or NT-proBNP ≤400 pg/mL	247 (29.7)	294 (15.5)	40 (13.8)	501 (20.5)
100< BNP ≤200 or 400< NT-proBNP ≤900 pg/mL	100 (12.0)	313 (16.5)	42 (14.5)	371 (15.2)
200< BNP ≤300 or 900< NT-proBNP ≤2000 pg/mL	79 (9.5)	261 (13.7)	44 (15.2)	296 (12.1)
BNP >300 or NT-proBNP ≥2000 pg/mL	240 (28.8)	673 (35.4)	134 (46.2)	779 (31.9)
Missing	167 (20.0)	361 (19.0)	30 (10.3)	498 (20.4)
Comorbidity, n (%)				
Hypertension	717 (86.1)	1,648 (86.6)	243 (83.8)	2,122 (86.8)
Chronic kidney disease	421 (50.5)	1,164 (61.2)	159 (54.8)	1,426 (58.3)
Ischemic heart disease	472 (56.7)	1,067 (56.1)	165 (56.9)	1,374 (56.2)
Atrial fibrillation	306 (36.7)	848 (44.6)	132 (45.5)	1,022 (41.8)
Diabetes mellitus	340 (40.8)	614 (32.3)	101 (34.8)	853 (34.9)
Stroke	152 (18.2)	417 (21.9)	63 (21.7)	506 (20.7)
Myocardial infarction	184 (22.1)	312 (16.4)	61 (21.0)	435 (17.8)
Chronic obstructive pulmonary disease	118 (14.2)	398 (20.9)	63 (21.7)	453 (18.5)
Anemia	249 (29.9)	660 (34.7)	124 (42.8)	785 (32.1)
Hyperkalemia	83 (10.0)	221 (11.6)	28 (9.7)	276 (11.3)
Hypotension	19 (2.3)	35 (1.8)	11 (3.8)	43 (1.8)
HF treatments prior to the index date,** n (%)				
ACEi	135 (16.2)	241 (12.7)	49 (16.9)	327 (13.4)
ARB	347 (41.7)	728 (38.3)	65 (22.4)	1,010 (41.3)
MRA	157 (18.8)	304 (16.0)	50 (17.2)	411 (16.8)
Beta-blockers	358 (43.0)	700 (36.8)	118 (40.7)	940 (38.4)
SGLT-2i	25 (3.0)	34 (1.8)	6 (2.1)	53 (2.2)
Digoxin/ digitoxin	43 (5.2)	98 (5.2)	8 (2.8)	133 (5.4)
Loop diuretics	406 (48.7)	1,049 (55.2)	155 (53.4)	1,300 (53.2)
Thiazide diuretics	57 (6.8)	166 (8.7)	16 (5.5)	207 (8.5)
Tolvaptan	49 (5.9)	97 (5.1)	18 (6.2)	128 (5.2)

SD, standard deviation; BNP, brain natriuretic peptide; NT-proBNP, N-terminal pro-brain natriuretic peptide; ACEi, angiotensin-converting enzyme inhibitor; ARB, angiotensin-receptor blocker; MRA, mineralocorticoid receptor antagonist; SGLT-2i, sodium-glucose cotransporter-2 inhibitor; HF, heart failure.

* Hospitalization for HF occurred within 12 months before the index date of hospitalization. ** Used during the period of 183 days before the index date.

Table S6. Sensitivity analysis of the proportions of days covered in all patients and in subgroups stratified based on age and a history of prior hospitalization for heart failure assessed based on the 60-day prescription gaps for discontinuation episodes.

	Overall	<75 years	≥75 years	With prior HF hospitalization*	No prior HF hospitalization*
Proportions of days covered,** mean ± SD					
ACEi	0.72 ± 0.36	0.77 ± 0.33	0.69 ± 0.37	0.67 ± 0.35	0.73 ± 0.36
ARB	0.67 ± 0.37	0.79 ± 0.32	0.59 ± 0.38	0.67 ± 0.35	0.67 ± 0.37
MRA	0.65 ± 0.37	0.70 ± 0.37	0.62 ± 0.37	0.58 ± 0.37	0.65 ± 0.37
Beta-blockers	0.71 ± 0.36	0.78 ± 0.34	0.65 ± 0.37	0.67 ± 0.37	0.71 ± 0.36
SGLT-2i	0.78 ± 0.32	0.79 ± 0.32	0.76 ± 0.31	0.76 ± 0.36	0.78 ± 0.32
Digoxin/ digitoxin	0.60 ± 0.36	0.63 ± 0.36	0.59 ± 0.37	0.72 ± 0.34	0.59 ± 0.37
Loop diuretics	0.71 ± 0.36	0.71 ± 0.35	0.70 ± 0.36	0.66 ± 0.37	0.71 ± 0.36
Thiazide diuretics	0.59 ± 0.37	0.64 ± 0.37	0.56 ± 0.37	0.52 ± 0.40	0.59 ± 0.37
Tolvaptan	0.74 ± 0.35	0.72 ± 0.35	0.75 ± 0.35	0.67 ± 0.38	0.75 ± 0.35

SD, standard deviation; ACEi, angiotensin-converting enzyme inhibitor; ARB, angiotensin-receptor blocker; MRA, mineralocorticoid receptor antagonist; SGLT-2i, sodium-glucose cotransporter-2 inhibitor; HF, heart failure.

* Hospitalization for HF occurred within 12 months before the index date of hospitalization.

** The analyses were performed in patients who could be followed up for 365 days after the initiation of any HF treatment assessed in the study. the treatment discontinuation was assessed based on the absence of a continuous prescription record of the HF treatment of interest for 60 days.

Fig. S1. Kaplan–Meier curves of all-cause death and re-hospitalization for HF. Panel (A) and panel (B) show Kaplan–Meier curves of all-cause death and re-hospitalization for HF, respectively.

(A)

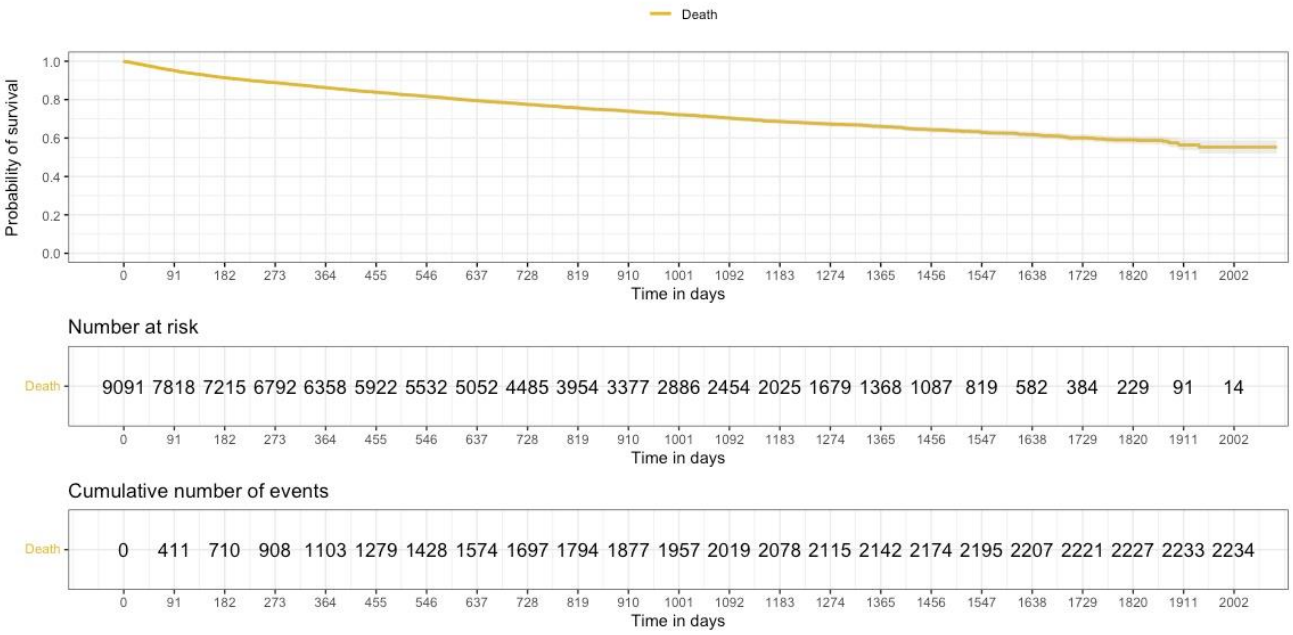

(B)

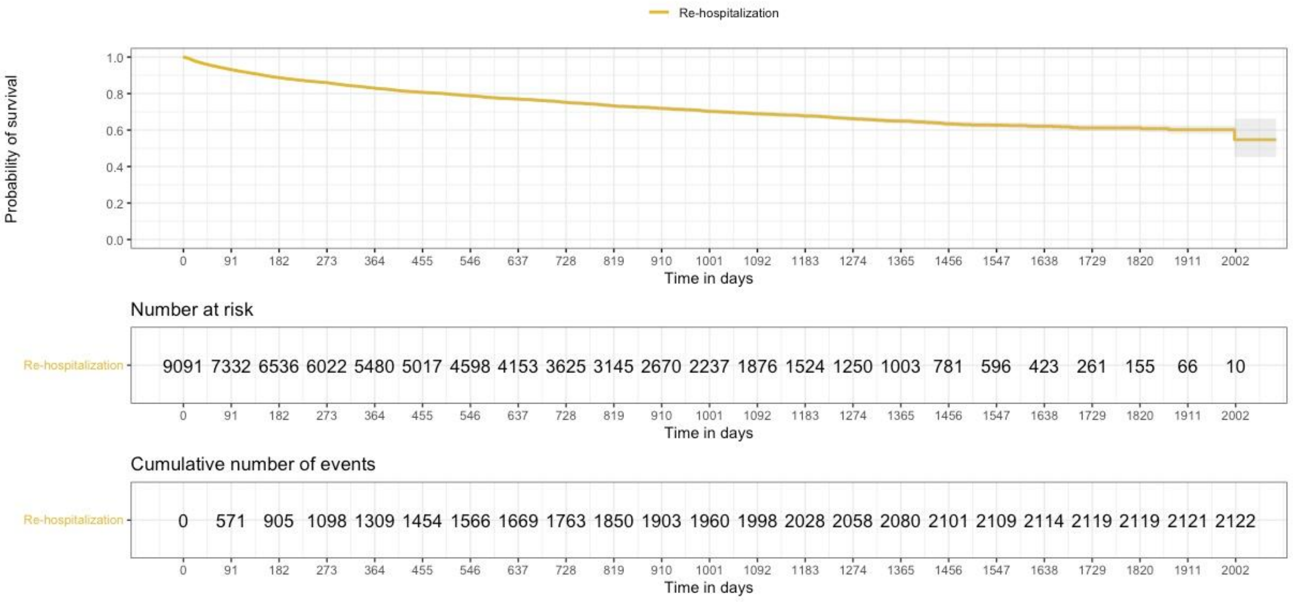

Fig. S2. Sensitivity analyses of continuous use of HF medications during the first year after initiation based on the 60-day prescription gaps for treatment discontinuation episodes. The analysis was performed in patients who could be followed up for 365 days after the initiation of any HF treatment assessed in each new-user cohort. Abbreviations: ACEi, angiotensin-converting-enzyme inhibitor; ARB, angiotensin receptor blocker; HF, heart failure; MRA, mineralocorticoid receptor antagonist; and SGLT-2i, sodium-glucose co-transporter-2 inhibitors

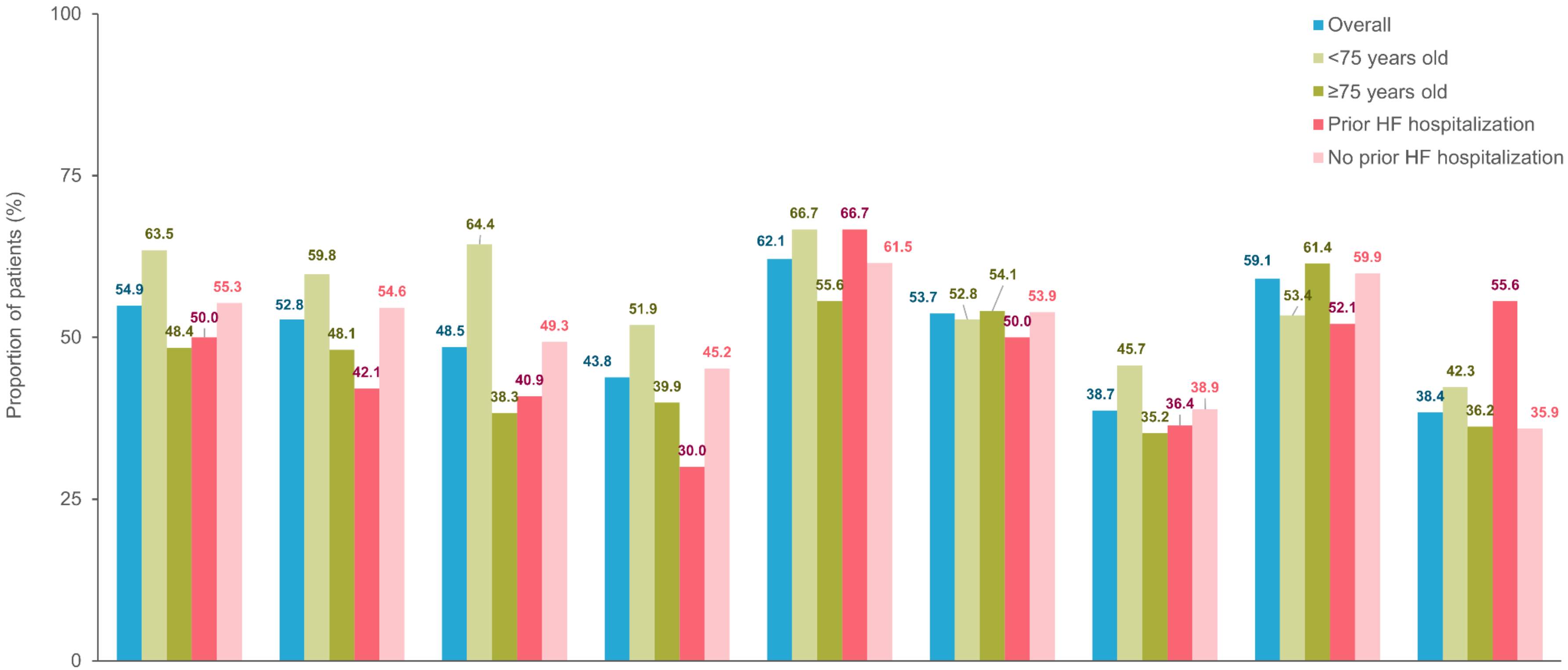

N of patients	Beta-blockers	ACEi	ARB	MRA	SGLT2i	Loop diuretics	Thiazide diuretics	Tolvaptan	Digoxin/Digitoxin
Overall	545	267	231	566	87	557	137	467	73
<75 years old	233	107	90	183	51	180	46	133	26
≥75 years old	312	160	141	383	36	377	91	334	47
Prior HF hosp.	42	38	22	50	9	34	11	48	9
No prior HF hosp.	503	229	209	516	78	523	126	419	64

References

1. Levey AS, Eckardt K-U, Tsukamoto Y, Levin A, Coresh J, Rossert J, et al. Definition and classification of chronic kidney disease: a position statement from Kidney Disease: Improving Global Outcomes (KDIGO). *Kidney Int.* 2005; 67 (6): 2089-100.

2. von Elm E, Altman DG, Egger M, Pocock SJ, Gøtzsche PC, Vandenbroucke JP, et al. The Strengthening the Reporting of Observational Studies in Epidemiology (STROBE) Statement: guidelines for reporting observational studies. *Ann Int Med.* 2008; 148(2): 573-577.
